# Deep learning-based pseudo-CT synthesis from zero echo time MR sequences of the pelvis

**DOI:** 10.1186/s13244-024-01751-3

**Published:** 2024-08-09

**Authors:** Jonas M. Getzmann, Eva Deininger-Czermak, Savvas Melissanidis, Falko Ensle, Sandeep S. Kaushik, Florian Wiesinger, Cristina Cozzini, Luca M. Sconfienza, Roman Guggenberger

**Affiliations:** 1 Institute of Diagnostic and Interventional Radiology, University Hospital Zurich (USZ), Zurich, Switzerland; 2 University of Zurich (UZH), Zurich, Switzerland; 3 Unit of Diagnostic and Interventional Radiology, IRCCS Istituto Ortopedico Galeazzi, Milan, Italy; 4 Institute of Forensic Medicine, University of Zurich (UZH), Zurich, Switzerland; 5 GE HealthCare, Oskar-Schlemmer-Strasse 11, Munich, Germany; 6 University of Milan, Department of Biomedical Sciences for Health, Milan, Italy

**Keywords:** Artificial intelligence, Deep learning, Synthetic computed tomography, Zero echo time, Magnetic resonance imaging

## Abstract

**Objectives:**

To generate pseudo-CT (pCT) images of the pelvis from zero echo time (ZTE) MR sequences and compare them to conventional CT.

**Methods:**

Ninety-one patients were prospectively scanned with CT and MRI including ZTE sequences of the pelvis. Eleven ZTE image volumes were excluded due to implants and severe B1 field inhomogeneity. Out of the 80 data sets, 60 were used to train and update a deep learning (DL) model for pCT image synthesis from ZTE sequences while the remaining 20 cases were selected as an evaluation cohort. CT and pCT images were assessed qualitatively and quantitatively by two readers.

**Results:**

Mean pCT ratings of qualitative parameters were good to perfect (2–3 on a 4-point scale). Overall intermodality agreement between CT and pCT was good (ICC = 0.88 (95% CI: 0.85–0.90); *p* < 0.001) with excellent interreader agreements for pCT (ICC = 0.91 (95% CI: 0.88–0.93); *p* < 0.001). Most geometrical measurements did not show any significant difference between CT and pCT measurements (*p* > 0.05) with the exception of transverse pelvic diameter measurements and lateral center-edge angle measurements (*p* = 0.001 and *p* = 0.002, respectively). Image quality and tissue differentiation in CT and pCT were similar without significant differences between CT and pCT CNRs (all *p* > 0.05).

**Conclusions:**

Using a DL-based algorithm, it is possible to synthesize pCT images of the pelvis from ZTE sequences. The pCT images showed high bone depiction quality and accurate geometrical measurements compared to conventional CT.

**Critical relevance statement:**

pCT images generated from MR sequences allow for high accuracy in evaluating bone without the need for radiation exposure. Radiological applications are broad and include assessment of inflammatory and degenerative bone disease or preoperative planning studies.

**Key Points:**

- pCT, based on DL-reconstructed ZTE MR images, may be comparable with true CT images.
- Overall, the intermodality agreement between CT and pCT was good with excellent interreader agreements for pCT.
- Geometrical measurements and tissue differentiation were similar in CT and pCT images.

**Graphical Abstract:**

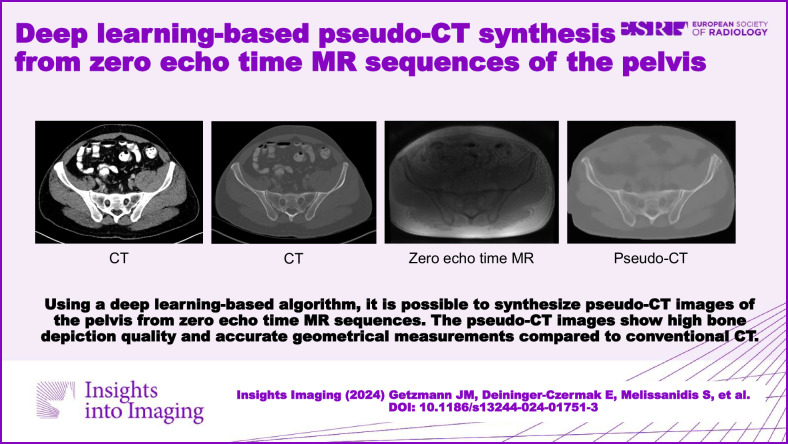

## Introduction

Pseudo-CT (pCT) imaging enables the generation of CT images from MR scans [1] which can be valuable for detecting early inflammatory and degenerative changes in the spine and hip joints [2, 3], as well as for preoperative examinations in spine surgery [4] or pre-partum examinations in gynecology [5]. Furthermore, the scaled density information of Hounsfield units (HU) available in CT or pCT is helpful for distinguishing bones from soft tissues or muscles from fat, especially in terms of radiation planning or body composition profiling (such as in sarcopenia or myosteatosis) [6–8].

While MRI is perfect for the evaluation of bone marrow and soft tissues, it has disadvantages in pure bone detection [9]. Advanced MR bone imaging sequences such as ultrashort echo time or zero echo time (ZTE) sequences can increase specificity in this regard [10, 11], but do not allow for quantitative attenuation (HU) information for bone and soft tissues like CT. Deep learning (DL) based on bone-specific ZTE MR images potentially allows for both high accuracy in bone geometry and efficient simulation of soft tissue contrast of fat and muscle, including specific scaled X-ray attenuation in the form of HU.

The aim of this study was to investigate the comparability of pCT images of the pelvis based on DL-reconstructed ZTE MR images with true CT images, in terms of both qualitative or geometric accuracy and simulated HU-based scaled X-ray attenuation for bone and soft tissues.

## Methods

This prospective study was approved by the institutional review board and the local ethics committee. Written informed consent was obtained from all participants prior to study inclusion. The study employs imaging data from a previously published cohort [2]. The prior report compared bone assessment of the sacroiliac (SI) joint by CT and ZTE MRI. The current study expands this by generating DL-based pCT images from the acquired ZTE MR sequences.

### Study participants

Individuals aged > 18 years who were referred for clinically indicated MR scans of the abdomen or pelvis between May 2019 and January 2021 were recruited. All patients considered for enrollment had undergone a CT scan covering the SI joints within 12 months of their MR examination. If patients agreed to participate in the study, an additional ZTE sequence was added to the respective standard MR protocol.

Exclusion criteria were refusal to participate in the study, pregnancy, contra-indications to MRI, any form of incomplete datasets, major artifacts due to motion or foreign bodies, or severe B1 field inhomogeneity.

### MR imaging

All MR scans were obtained with a 3.0-T scanner (Discovery 750W plus GEM; GE Healthcare) using an abdominal coil. Different protocols were used according to the respective clinical indications. At the end of each protocol, the same ZTE sequence was acquired in each participant (oZTEo, GE Healthcare; TR, 5.1 ms; TE, ≈ 0 ms; acquisition matrix, 212 × 212 × 250; slice thickness, 1.5 mm; field of view, 320 mm; bandwidth, ± 62.5 kHz; flip angle, 2°; scan time, 4:06 min). ZTE images were acquired in an axial plane in isotropic resolution. The typical through-plane coverage was 250 slices, ranging from the 12th thoracic vertebra to the lesser trochanter.

### pCT synthesis

pCT images were generated from ZTE MRI using the method previously described in [12]. The solution consists of a 2D multi-layer convolution neural network adapted to a multi-task UNet framework. The network is designed to maintain the overall structural accuracy of the image while focusing on achieving precise bone representation by learning correlated tasks: (a) image translation as the primary task, (b) bone segmentation, and (c) bone density value estimations as auxiliary tasks. Each task is optimized individually using a dedicated loss function that is customized to minimize a specific error, and the combined loss value contributes towards the overall training of the network. By separating the tasks of classification and regression, and by optimizing the network to reduce both errors simultaneously, implicit reinforcement can be achieved towards each of the correlated tasks [13]. Although the tasks are correlated, the network is expected to learn them differently from one another.

### CT imaging

CT examinations were performed on different scanners. The majority of scans consisted of an abdominal or pelvic CT obtained with the latest generation energy-integrating dual-source scanner (Somatom Definition FLASH, Siemens Healthineers; tube voltage, 90 kVp, 100 kVp, 110 kVp, and 120 kVp; tube current, 100–150 mAs with active tube modulation); field of view, 500 mm with a matrix of 512 × 512; bone kernel (mostly Br59) for axial image reconstruction). Image data were then reformatted in all three planes with a slice thickness and increment of 1.5 mm, each.

### Image analysis

The assessment of CT and pCT images was performed independently by two fellows in musculoskeletal radiology (J.M.G. and S.M., both 5 years of experience) after careful instructions by a senior musculoskeletal radiologist with more than 15 years of experience (R.G.). The readout of the two datasets (CT and pCT, respectively) was conducted in two different sessions, separated by 4 weeks and in different random order to avoid recall bias. Both readers were blinded to patient identification and clinical data, as well as to the results of the other datasets. CT images were evaluated using the institution’s picture archiving and communication system (DeepUnity Diagnost, version R20 XX; Dedalus S.p.A.). pCT images were viewed using Synedra View 21 (version 21.0.0.8 (× 64 edition); Synedra Information Technologies GmbH).

#### Qualitative analysis

CT and pCT images were rated qualitatively using a 4-point Likert scale (0–3; 0 = poor, 1 = slight, 2 = good, and 3 = perfect) [14] with regard to the following parameters: sharpness of bone contour, differentiation of cortical and trabecular bone, delineation of hip joint space, delineation of SI joint space, and preservation of soft tissue boundaries. For pCT, body masking and severity of false bone classification around the pelvis (0–3; 0 = none, 1 = slight, 2 = marked, and 3 = severe) were assessed additionally. Subjective assessment confidence was rated separately for all image series (0–3; 0 = poor confidence that makes it almost impossible for assessment, 1 = low confidence that may affect the assessment, 2 = moderate confidence that does not affect the assessment negatively, and 3 = high confidence facilitating a clear assessment).

#### Quantitative analysis

To assess the geometrical accuracy of the synthesized pCT images, the following measurements were performed in both CT and pCT images: distance between the center of the femoral heads in the axial plane, transverse (greatest width of the superior pelvic aperture) and anteroposterior (measured from the pubic symphysis to the sacral promontory) pelvic diameter, alpha angle of the right femur in the oblique axial plane, and lateral center-edge angle of the right femur in the coronal plane.

Additionally, HU values (mean, SD, minimum, and maximum) were determined using same-sized region of interest (ROI) measurements (3 mm^2^ for cortical bone; 15 mm^2^ for all other anatomic locations) in the following structures in both CT and pCT: cortical bone of the body of right ilium, trabecular bone of the body of right ilium, right gluteus maximus, subcutaneous fat adjacent to right gluteus maximus, and air close to tissue in areas that were visually free of noise.

To quantitatively assess image quality and tissue differentiation, contrast-to-noise ratios (CNR) were calculated for CT and pCT images with the following formula:

$${{CNR}}\,=\,\frac{{{{\rm{|}}}}{{HU}}_{{{mean}}}(A)-{{{HU}}}_{{{mean}}}(B){{{\rm{|}}}}}{{{{HU}}}_{{{SD}}}{{air}}}$$

where (A) and (B) are structures in the ROI, and air is defined as pure image noise.

### Statistical analysis

All statistical analyses were conducted using SPSS (version 29.0; IBM). *p*-values < 0.05 were considered statistically significant. Intraclass correlation coefficients (ICC) were calculated for all qualitative categories based on Likert scales to assess rating consistencies between both readers and methods. ICC estimates and their 95% confidence intervals (CI) were calculated based on a mean rating (*k* = 2), consistency agreement, and a two-way mixed-effects model. ICC values less than 0.50 were considered poor, between 0.50 and 0.75 moderate, between 0.75 and 0.90 good, and above 0.90 as an excellent agreement [15]. All distance and angle measurements were first evaluated regarding their normal distribution using a Shapiro–Wilk test [16]. If a normal distribution was present, a paired sample *t*-test was applied to evaluate differences between readers, respectively methods. If measurements did not show a normal distribution, a Wilcoxon signed rank test was calculated and on all significant results, a post-hoc Holm–Bonferroni test for multiple comparisons was performed [17].

## Results

**Fig. 1:**
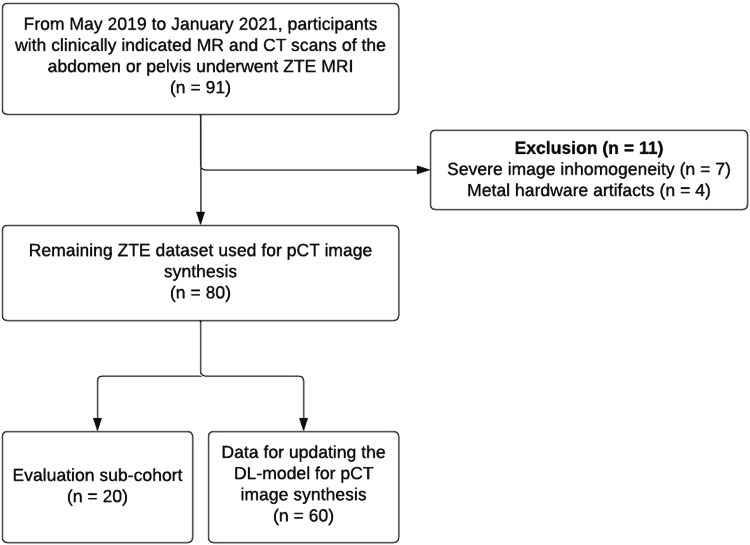
Flowchart for participant inclusion. DL, deep learning; pCT, pseudo-CT; ZTE, zero echo time

**Fig. 2:**
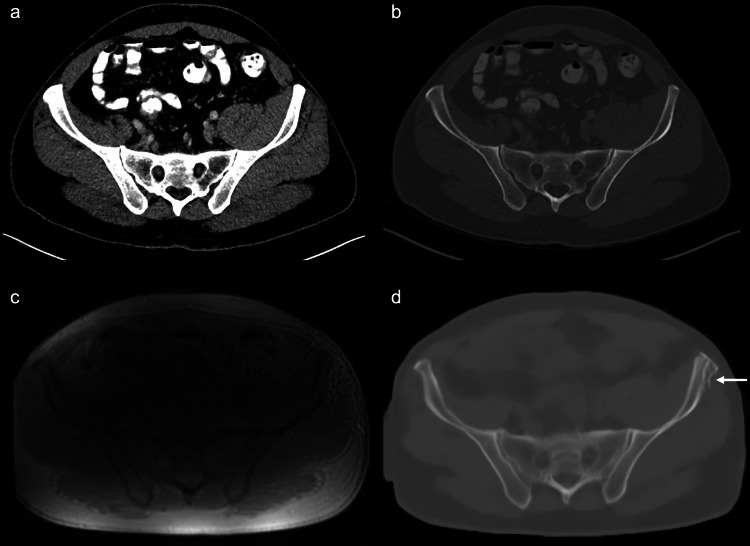
Exemplary CT (**a**, **b**), ZTE MRI (**c**), and pCT (**d**) images of the pelvis of a 45-year-old study participant. The pCT (**d**) image was generated from ZTE MRI (**c**) by applying a deep-learning algorithm. Note the almost perfect body masking and the small area that was falsely classified as “bone” (arrow) in (**d**)

**Fig. 3:**
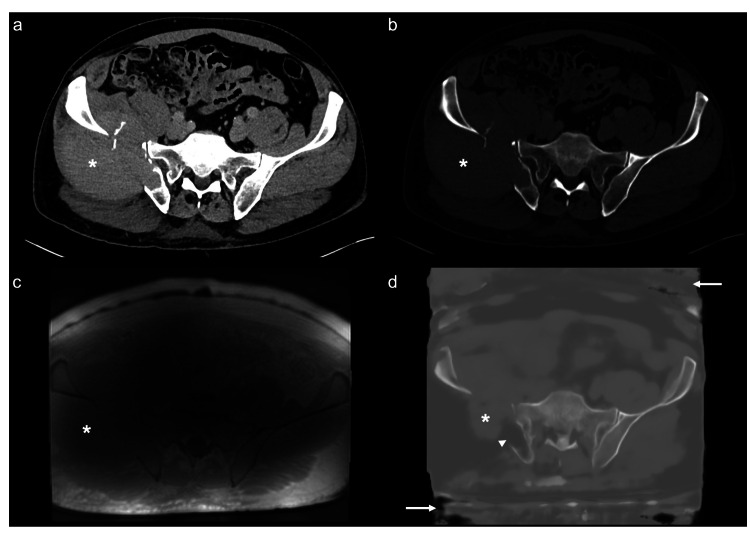
Exemplary CT (**a**, **b**), ZTE MRI (**c**), and pCT (**d**) images of the pelvis of a 54-year-old study participant. The pCT (**d**) image was generated from ZTE MRI (**c**) by applying a deep-learning algorithm. The images display a large tumor in the right iliac wing (asterisk; biopsy-proven plasmacytoma) which was underestimated in **d** since the model was trained with enhanced focus on bone regions. Nevertheless, lytic bone destruction by the tumor was correctly identified (arrowhead). Note the imperfect body masking (arrows) in **d**

Participant inclusion is summarized in Fig. 1. A summary of participant characteristics is shown in Table 1. A total of 91 participants (58 men, 33 women, age 56 ± 15 years (mean ± SD), range 20–86 years) were prospectively recruited and scanned with MRI including ZTE sequences. Eleven ZTE image volumes were excluded due to poor image quality because of severe image inhomogeneity (*n* = 7; 64%) or metal hardware artifacts (*n* = 4; 36%). Out of the 80 remaining data sets, 20 patient cases (12 men, 8 women, age 46 ± 14 years (mean ± SD), range 20–72 years) were selected as an evaluation cohort (= validation data set) and the remaining data was used to train and update the existing DL-model for pCT image synthesis from ZTE sequences (= training data set). Exemplary CT and pCT images of the pelvis are shown in Figs. 2 and 3.

**Table 1:** Characteristics of the study participants and the evaluation sub-cohort

Characteristic	Value	
	All study participants, (*n* = 91)	Evaluation sub-cohort, (*n* = 20)
Age (years)	56 ± 15 (20–86)	46 ± 14 (20–72)
Sex^a^		
Male	58	12
Female	33	8
Time between CT and MRI (months)	5.4 ± 4.3 (0–12)	6.2 ± 4.7 (0–12)
Clinical indication for CT and MRI^b^		
Tumor	57.1 (52/91)	45 (9/20)
Infectious	12.1 (11/91)	15 (3/20)
Chronic musculoskeletal disorders	9.9 (9/91)	15 (3/20)
Trauma	5.5 (5/91)	0
Abdominal/inguinal hernia	5.5 (5/91)	15 (3/20)
Unknown diagnosis	9.9 (9/91)	10 (2/20)

Unless otherwise indicated, data are mean ± standard deviation, with the range in parentheses

^a^ Data is the number of participants

^b^ Data is percentages, with the numerator and denominator in parentheses

### Qualitative analysis

An overview of qualitative results is given in Table 2. Mean CT and pCT ratings for the sharpness of bone contour, differentiation of cortical and trabecular bone, delineation of hip joint space, delineation of SI joint space, and preservation of soft tissue boundaries were good (2) to perfect (3), however, CT received significantly higher ratings than pCT by both readers in all categories (see Table 2 for *p*-values). The mean rating for body masking was perfect for CT (3.00 ± 0 (mean ± SD)) and slight to good for pCT (1.58 ± 0.75, range 1–3; *p* < 0.001). There were no false bone classifications around the pelvis in CT. In pCT, the false bone classifications were slightly to marked (1.38 ± 0.59, range 1–3; *p* < 0.001). Mean subjective assessment confidence was perfect for CT (3.00 ± 0) and good to perfect for pCT (2.70 ± 0.46, range 2–3; *p* = 0.001). Post-hoc Holm–Bonferroni tests for multiple comparisons confirmed the significant results.

**Table 2:** Intermodality assessment of qualitative ratings of CT and pCT by both readers

Qualitative parameter	CT, (*n* = 40)	pCT, (*n* = 40)	*p*-value
Sharpness of bone contour	2.98 ± 0.16 (2–3)	2.63 ± 0.49 (2–3)	< 0.001
Differentiation of cortical and trabecular bone	3.00 ± 0 (3–3)	2.88 ± 0.34 (2–3)	0.03
Delineation of hip joint space	2.87 ± 0.34 (2–3)	2.28 ± 0.62 (1–3)	< 0.001
Delineation of SI joint space	2.88 ± 0.34 (2–3)	2.53 ± 0.56 (1–3)	0.001
Preservation of soft tissue boundaries	3.00 ± 0 (3–3)	2.23 ± 0.70 (1–3)	< 0.001
Body masking	3.00 ± 0 (3–3)	1.58 ± 0.75 (1–3)	< 0.001
Severity of false bone classification around the pelvis	0.00 ± 0 (0–0)	1.38 ± 0.59 (1–3)	< 0.001
Subjective assessment confidence	3.00 ± 0 (3–3)	2.70 ± 0.46 (2–3)	0.001

Unless otherwise indicated, data are mean ± standard deviation, with the range in parentheses. The parameters were rated with a 4-point Likert scale (for the severity of false bone classification: 0 = none, 1 = slight, 2 = marked, and 3 = severe; for all other parameters: 0 = poor, 1 = slight, 2 = good, and 3 = perfect)

*pCT* indicates pseudo-CT, *SI* sacroiliac

Overall intermodality agreement for qualitative assessment parameters between CT and pCT was good (ICC = 0.88 (95% CI: 0.85–0.90); *p* < 0.001). The overall interreader agreement was excellent (ICC = 0.95 (95% CI: 0.94–0.96); *p* < 0.001) with excellent interreader agreements for CT (ICC = 0.99 (95% CI: 0.98–0.99); *p* < 0.001) and for pCT (ICC = 0.91 (95% CI: 0.88–0.93); *p* < 0.001).

### Quantitative analysis

**Fig. 4:**
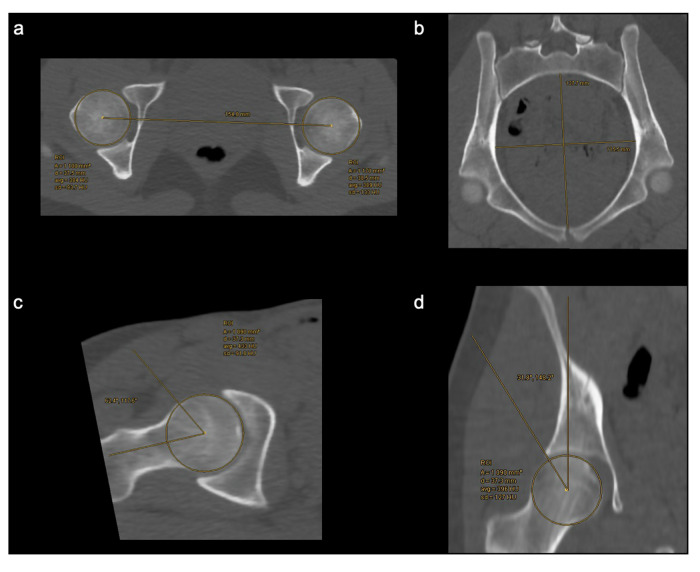
Illustrative measurements for quantitative analysis on CT images of a 20-year-old study participant. **a** Distance between the center of the femoral heads in the axial plane, (**b**) transverse (greatest width of the superior pelvic aperture) and anteroposterior (measured from the pubic symphysis to the sacral promontory) pelvic diameter, (**c**) alpha angle of the right femur in the oblique axial plane, and (**d**) lateral center-edge angle of the right femur in the coronal plane

**Fig. 5:**
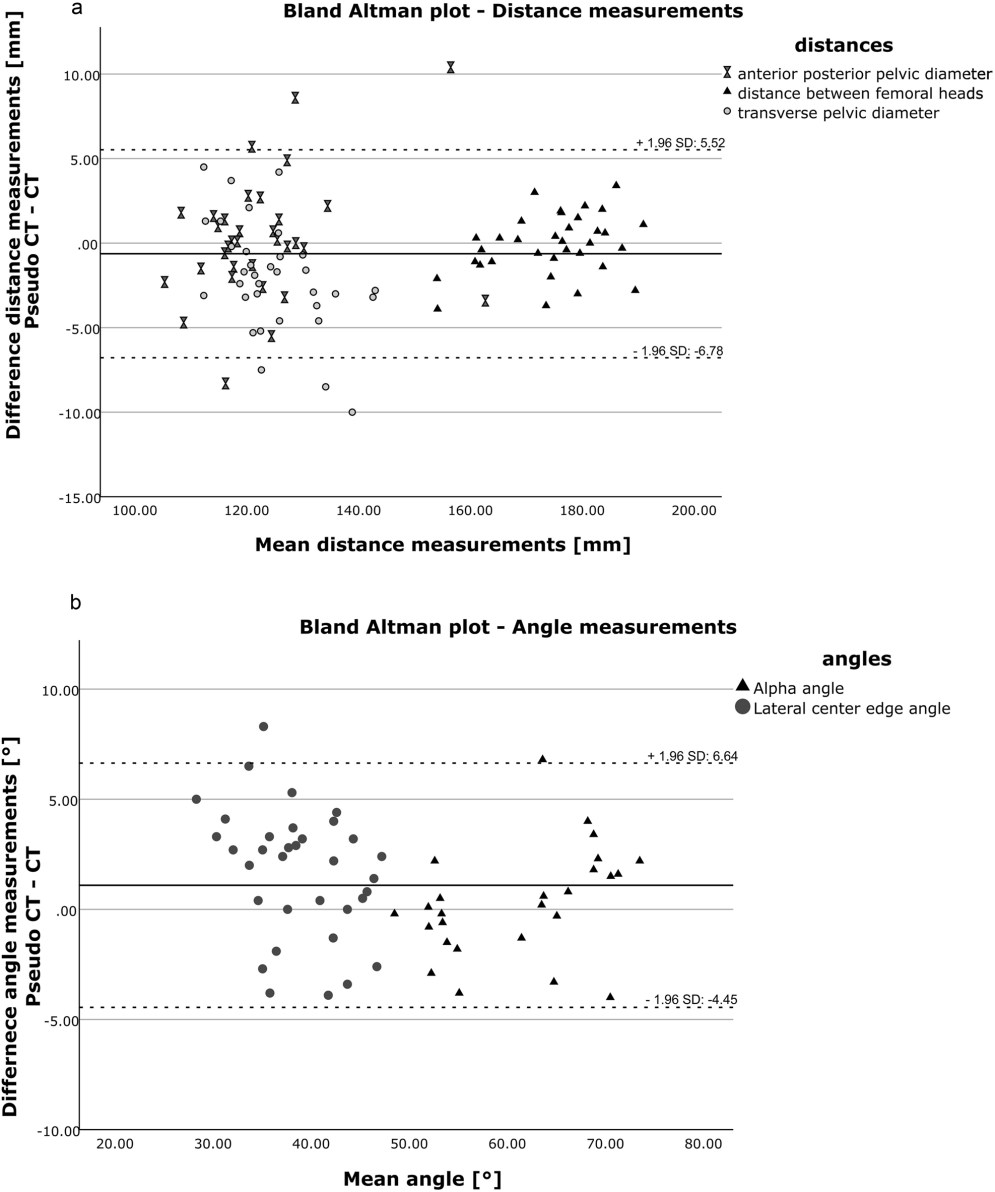
Bland Altman plots showing measurement variances between pCT and CT. Differences were calculated from paired measurements conducted in the same case. **a** Distance measurements of pelvic diameters did show, except for singular outliers, differences of less than 5 mm. Of all measured variables the distance between the centers of the femoral heads showed the highest concordance, possibly due to the most comprehensive anatomical landmark. **b** Angle measurements proportionally showed a higher deviation between measurements, however no trend towards higher values in either imaging method was observed. This may be explained by a more substantial influence of individual measurement deviations

Distance and angle measurements in CT and pCT are shown illustratively in Fig. 4 and summarized in Table 3. Transverse pelvic diameter measurements and lateral center-edge angle measurements of the right femur were significantly different between CT and pCT (*p* = 0.001 and *p* = 0.002, respectively) while mean differences were relatively small (1.98 mm and 1.71°, respectively). All other geometrical measurements were not significantly different between CT and pCT (all *p* > 0.05). CT and pCT alpha angle measurements of the right femur were significantly different between the two readers (both *p* < 0.001; mean difference 13.67° in CT and 14.28° in pCT); smaller significant interreader differences could be observed in CT transverse pelvic diameter measurements (*p* = 0.03; mean difference 2.57 mm) and in CT lateral center-edge angle measurements of the right femur (*p* = 0.03; mean difference 2.30°). Except for pCT alpha angle measurements of the right femur, there were no significant differences between readers in pCT distance and angle measurements (Fig. 5a, b).

**Table 3:** Interreader and intermodality assessments of distance and angle measurements in CT and pCT

Quantitative parameter		Interreader assessment			Intermodality assessment	
		Reader 1, (*n* = 20)	Reader 2, (*n* = 20)	*p*-value	Both readers, (*n* = 40)	*p*-value
Distance between the center of the femoral heads (mm)	CT	175.51 ± 9.07	175.82 ± 9.32	0.39	175.38 ± 9.36	0.47
	pCT	176.04 ± 11.53	176.67 ± 11.01	0.17	175.15 ± 9.89	
Transverse pelvic diameter (mm)	CT	128.08 ± 7.86	125.51 ± 9.32	**0.03**	126.55 ± 8.88	**0.001**
	pCT	125.63 ± 7.93	124.50 ± 7.69	0.17	124.57 ± 7.38	
Antero-posterior pelvic diameter (mm)	CT	125.94 ± 12.45	123.35 ± 10.89	0.06	123.04 ± 11.08	0.73
	pCT	123.68 ± 11.87	122.95 ± 12.80	0.51	123.32 ± 12.16	
Alpha angle right femur (°)	CT	67.77 ± 3.35	54.10 ± 5.14	**<** **0.001**	61.58 ± 7.19	0.51
	pCT	68.74 ± 3.84	54.46 ± 4.53	**<** **0.001**	61.89 ± 8.11	
Lateral center-edge angle right femur (°)	CT	38.16 ± 6.61	40.46 ± 7.78	**0.03**	38.22 ± 5.74	**0.002**
	pCT	40.29 ± 4.27	39.58 ± 5.34	0.41	39.93 ± 4.77	

Unless otherwise indicated, data are mean ± standard deviation

*pCT* indicates pseudo-CT

Bold values indicate statistical significance *p*-values < 0.05

HU measurements of different structures in CT and pCT are shown in Table 4. All HU measurements were significantly higher in CT than in pCT (all *p* < 0.05). HU measurements were significantly different in the cortical bone of the body of the right ilium in CT (*p* = 0.001) and in the muscle of the right gluteus maximus in pCT (*p* = 0.003). All other HU measurements were not significantly different between the two readers.

**Table 4:** Interreader and intermodality assessments of HU measurements in CT and pCT

Structure		Interreader assessment			Intermodality assessment	
		Reader 1, (*n* = 20)	Reader 2, (*n* = 20)	*p*-value	Both readers, (*n* = 40)	*p*-value
Cortical bone of the body of right ilium (HU)	CT	1326.00 ± 188.52	1186.80 ± 131.64	**0.001**	1256.40 ± 175.29	**<** **0.001**
	pCT	822.63 ± 73.68	812.36 ± 64.72	0.56	817.49 ± 68.65	
Trabecular bone of the body of right ilium (HU)	CT	252.82 ± 98.41	266.90 ± 77.91	0.41	259.86 ± 87.90	**<** **0.001**
	pCT	181.38 ± 47.44	188.29 ± 42.96	0.66	184.83 ± 44.81	
Right gluteus maximus (HU)	CT	53.99 ± 10.98	54.21 ± 11.29	0.91	54.10 ± 10.99	**<** **0.001**
	pCT	21.17 ± 5.63	24.27 ± 5.27	**0.003**	22.72 ± 5.61	
Subcutaneous fat adjacent to right gluteus maximus (HU)	CT	−103.72 ± 11.10	−106.33 ± 10.16	0.16	−105.03 ± 10.59	**0.009**
	pCT	−111.31 ± 10.58	−113.15 ± 12.83	0.20	−112.23 ± 11.65	

Unless otherwise indicated, data are mean ± standard deviation

*HU* indicates Hounsfield unit, *pCT* pseudo-CT

Bold values indicate statistical significance *p*-values < 0.05

**Fig. 6:**
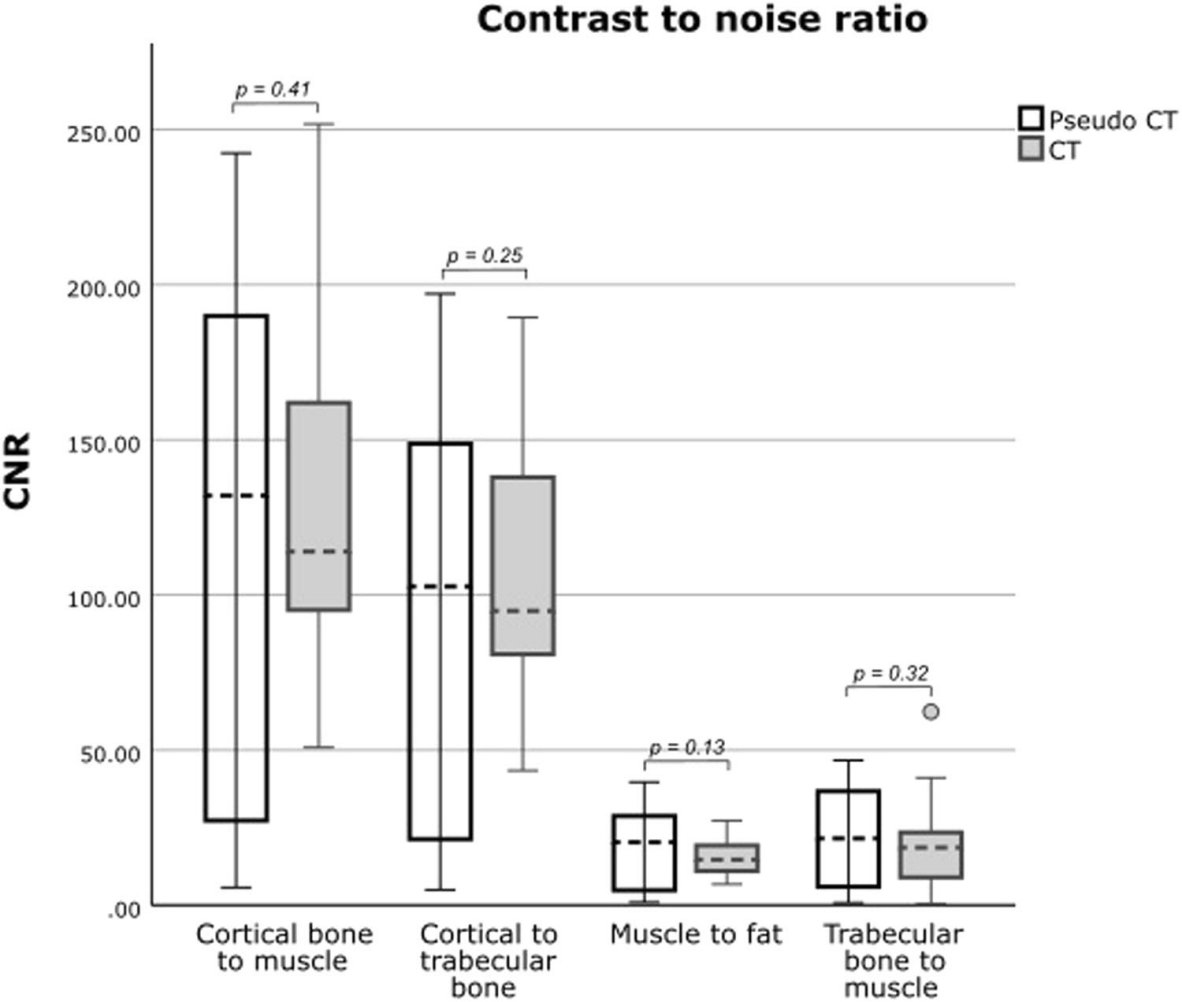
CNRs in CT and pCT images. pCT CNRs showed a bigger range of values compared to CT CNRs, however, mean values were similar and there was no significant difference between pCT and CT CNRs (all *p* > 0.05)

CNRs are shown in Table 5. pCT CNRs showed a bigger range of values compared to CT CNRs, however, mean values were similar and there was no significant difference between pCT and CT CNRs (all *p* > 0.05; Fig. 6).

**Table 5:** CNRs calculated in CT and pCT images of the pelvis

CNR	CT, (*n* = 20)	pCT, (*n* = 20)	*p*-value
CNR trabecular bone—muscle	19.41 ± 14.14	23.05 ± 16.54	0.50
CNR cortical bone—trabecular bone	105.10 ± 43.32	96.32 ± 66.10	0.63
CNR cortical bone—muscle	124.51 ± 50.86	119.36 ± 80.40	0.82
CNR muscle—fat	15.42 ± 5.90	19.15 ± 12.65	0.26

Unless otherwise indicated, data are mean ± standard deviation

*CNR* contrast-to-noise ratio

## Discussion

In this study, pCT images of the pelvis were generated from ZTE MR sequences and compared to conventional CT with regard to qualitative and quantitative assessment parameters. Mean pCT ratings of qualitative parameters were good to perfect (2–3 on the 4-point Likert scale). Overall intermodality agreement between CT and pCT was good (ICC = 0.88 (95% CI: 0.85–0.90); *p* < 0.001) with excellent interreader agreements for pCT (ICC = 0.91 (95% CI: 0.88-0.93); *p* < 0.001). Most geometrical measurements did not show any significant difference between CT and pCT measurements (*p* > 0.05) with the exception of transverse pelvic diameter measurements and lateral center-edge angle measurements. Image quality and tissue differentiation in CT and pCT were similar without significant differences between CT and pCT CNRs (all *p* > 0.05).

Previous studies have investigated the use of advanced MR sequences and pCT in bone imaging. Jans et al [3] used MR-based pCT images to detect structural lesions in patients with suspected sacroiliitis and found that pCT performed better than routine T1-weighted MRI. Argentieri et al [18] and Breighner et al [19] presented CT-like images based on ZTE MR sequences and reported good agreement with CT of the spine and hips. However, those sequences were lacking information on tissue density and could thus provide only qualitative image information. By generating pCT images from ZTE MR sequences, our study integrated qualitative image information of an advanced MR bone imaging sequence with DL-facilitated simulation of quantitative HU attenuation maps. This could also lead to new ways of assessing bone quantity and quality using HU values, through correlation with bone mineral density and trabecular bone strength. HU values are positively correlated with material density and compressive strength [20, 21]. The use of pCT images derived from ZTE MR sequences may be useful to give an idea of bone status opportunistically, in cases where morphological MR sequences may show stress or insufficiency fractures, for example.

Even though qualitative ratings of CT images were significantly higher than those of pCT images, absolute mean values of qualitative pCT ratings were still good to perfect in all assessment categories, leading to high subjective assessment confidence of the readers. Similarly, some of the geometrical measurements were significantly different between readers and modalities. When considering absolute mean values of transverse pelvic diameter one can observe however that the mean difference between the two modalities was less than 2 mm which could easily be due to measurement inaccuracy and would most likely be insignificant in clinical settings. The same observation was made when comparing mean values of lateral center-edge angle measurements between CT and pCT where the mean difference between the two modalities was less than 2°. Previously, angle measurements with a difference of less than 5° between readers have been considered identical [22]. The significant interreader difference for alpha angle measurements was most likely due to a systematic measurement error of one of the readers since there was no significant difference in intermodality assessment.

pCT HU values were consistently lower compared to CT HU values. The reason for the general underestimation of high-density HU values is mostly due to the sparsity of those regions in comparison to the overall image information. That is, the percentage of image regions (voxels) > 900 HU in CT is a tiny fraction of the body region. This sparsity gets overshadowed by the rest of the pixels and tends to approximate the learning to lower values. The authors believe that the difference between pCT and conventional CT values should become lower with further training of the algorithm with diverse data. On the other hand, pCT HU values allowed for correct tissue differentiation which is more important than absolute HU values in most clinical settings.

The authors acknowledge several limitations of this study. First, body masking for pCT remains challenging. This is mostly due to image inhomogeneity and attached external objects. To resolve this problem, additional background masking could be performed. In this study, the focus was however on deeper structures such as the pelvic bone and its adjacent soft tissues and body surface information was less important.

Second, there were high-density areas in soft tissues that were occasionally classified as bone in pCT images. Those areas were mostly located in muscles around the pelvic bones and could imitate calcifications. This was due to the DL model which was trained with an enhanced focus on bone regions, thus showing a fuller-appearing bone, but also resulting in some false bone classifications because of higher sensitivity towards high-density regions. In this context, it is important to notice that the ZTE data in this study were not enhanced by DL reconstructions. Nowadays, DL-reconstruction solutions for ZTE images are available and have been shown to enhance image quality [23, 24]. Therefore, higher DL-model performance in the generation of pCT images could be expected by employing DL-reconstructed ZTE images.

Third, metal artifacts remain difficult to overcome in MR imaging and therefore also represent a challenge in ZTE sequences and subsequently pCT images generated from ZTE sequences. In this study, participants with metal implants in and around the hips were excluded. The authors acknowledge that this may influence the generalizability of the study. In the future, further improvements with regard to the reduction of metal artifacts in MR imaging may make it possible to generate pCT images also in patients with metal implants.

Fourth, although HU and geometrical measurements were standardized, minor inconsistencies between readers during manual readout sessions might have persisted and impacted overall pCT accuracy.

In conclusion, it is possible to synthesize pCT images of the pelvis from ZTE sequences using a DL-based algorithm. The pCT images show high bone depiction quality and accurate quantitative measurements compared to conventional CT. Further training in the DL algorithm is necessary to improve body masking and reduce false bone classifications. Application of the algorithm in different body regions and in pathological settings (i.e., osteoarthritis and inflammatory bone disease) are objectives of future research.

### Supplementary information

ELECTRONIC SUPPLEMENTARY MATERIAL

## Data Availability

Data supporting the results reported in this article can be obtained from the corresponding author on request.

## Abbreviations

CI: Confidence interval
CNR: Contrast-to-noise ratio
DL: Deep learning
HU: Hounsfield unit
ICC: Intraclass coefficient
pCT: Pseudo-CT
ROI: Region of interest
SI: Sacroiliac
ZTE: Zero echo time
