# Supplementary material for: Deep learning-based pseudo-CT synthesis from zero echo time MR sequences of the pelvis
Source: Insights Imaging. 2024 Aug 9;15:202. doi: 10.1186/s13244-024-01751-3 (PMC11315823; doi:10.1186/s13244-024-01751-3)
Supplement: Supplementary file 1 — ELECTRONIC SUPPLEMENTARY MATERIAL [file 13244_2024_1751_MOESM1_ESM.pdf]

Deep learning-based pseudo-CT synthesis from zero echo time MR sequences of the pelvis

ELECTRONIC SUPPLEMENTARY MATERIAL

Insights Imaging (2024) Getzmann JM, Deininger-Czermak E, Melissanidis S, et al.

Checklist for Artificial Intelligence in Medical Imaging (CLAIM)

Deep learning-based pseudo-CT synthesis from zero echo time MR sequences of the pelvis

Checklist for Artificial Intelligence in Medical Imaging (CLAIM)

Section/Topic	No.	Item
TITLE or ABSTRACT		
	1	Identification as a study of AI methodology, specifying the category of technology used (eg, deep learning)
ABSTRACT		
	2	Structured summary of study design, methods, results, and conclusions
INTRODUCTION		
	3	Scientific and clinical background, including the intended use and clinical role of the AI approach
	4	Study objectives and hypotheses
METHODS		
Study Design	5	Prospective or retrospective study
	6	Study goal, such as model creation, exploratory study, feasibility study, noninferiority trial
Data	7	Data sources
	8	Eligibility criteria: how, where, and when potentially eligible participants or studies were identified (eg, symptoms, results from previous tests, inclusion in registry, patient-care setting, location, dates)
	9	Data preprocessing steps
	10	Selection of data subsets, if applicable
	11	Definitions of data elements, with references to common data elements
	12	De-identification methods
	13	How missing data were handled
Ground Truth	14	Definition of ground truth reference standard, in sufficient detail to allow replication
	15	Rationale for choosing the reference standard (if alternatives exist)
	16	Source of ground truth annotations; qualifications and preparation of annotators
	17	Annotation tools
	18	Measurement of inter- and intrarater variability; methods to mitigate variability and/or resolve discrepancies
Data Partitions	19	Intended sample size and how it was determined
	20	How data were assigned to partitions; specify proportions
	21	Level at which partitions are disjoint (eg, image, study, patient, institution)
Model	22	Detailed description of model, including inputs, outputs, all intermediate layers and connections
	23	Software libraries, frameworks, and packages
	24	Initialization of model parameters (eg, randomization, transfer learning)
Training	25	Details of training approach, including data augmentation, hyperparameters, number of models trained
	26	Method of selecting the final model
	27	Ensembling techniques, if applicable
Evaluation	28	Metrics of model performance
	29	Statistical measures of significance and uncertainty (eg, confidence intervals)
	30	Robustness or sensitivity analysis
	31	Methods for explainability or interpretability (eg, saliency maps) and how they were validated
	32	Validation or testing on external data
RESULTS		
Data	33	Flow of participants or cases, using a diagram to indicate inclusion and exclusion
	34	Demographic and clinical characteristics of cases in each partition
Model performance	35	Performance metrics for optimal model(s) on all data partitions
	36	Estimates of diagnostic accuracy and their precision (such as 95% confidence intervals)
	37	Failure analysis of incorrectly classified cases
DISCUSSION		
	38	Study limitations, including potential bias, statistical uncertainty, and generalizability
	39	Implications for practice, including the intended use and/or clinical role
OTHER INFORMATION		
	40	Registration number and name of registry
	41	Where the full study protocol can be accessed
	42	Sources of funding and other support; role of funders

Title

1. The category of AI technology is mentioned in the title: Deep learning-based pseudo-CT synthesis from zero echo time MR sequences of the pelvis.

Abstract

2. A structured summary of the study is provided.

Introduction

3. Scientific and clinical background, including the intended use and clinical role of the AI approach is provided.
4. Study objectives are stated at the end of the introduction: The aim of this study was to investigate the comparability of pseudo-CT images of the pelvis based on DL-reconstructed ZTE MR images with true CT images, in terms of both qualitative or geometric accuracy and simulated HU-based scaled X-ray attenuation for bone and soft tissues.

Methods

Study design

5. Prospective study.
6. Study goals: model creation, feasibility study, and noninferiority trial.

Data

7. Data sources: Imaging database of the Department of Diagnostic and Interventional Radiology at the University Hospital Zurich, Switzerland.
8. Eligibility criteria: Individuals aged >18 years who were referred for clinically indicated MR scans of the abdomen or pelvis between May 2019 and January 2021 were recruited. All patients considered for enrollment had undergone a CT scan covering the SI joints within 12 months of their MR examination.
9. Data preprocessing steps: The MR images were corrected for B1 inhomogeneity shading artifacts using N4 correction filter implemented in ITK toolkit and values were normalized using z-scores of each image. The CT images were retained in the native HU scale, with their values limited to the range [-1000,3000].
10. Selection of data subsets: The total available data were categorized into training, validation, and testing sets in the ration 70:20:10.
11. Definition of data elements: n.a.
12. De-identification methods: The images were de-identified to remove all patient health information and pseudo-anonymized subject IDs were used to identify the MR-CT image pairs corresponding to each subject.
13. Missing data: n.a.

Ground Truth

14. Definition of ground truth reference standard: A co-registered real CT image of each subject was considered as the reference ground truth for pseudo-CT model training for input MR images.
15. Rationale for choosing the reference standard: Real CT is considered as the reference standard for pseudo-CT image synthesis in the community based on current practices using CT images in clinical workflow applications.
16. Source of ground truth annotations: The CT images were aligned with MR images using non-rigid registration techniques implements in ANTs toolkit. The accuracy of registration was manually validated and misaligned regions within the images were discarded from training and validation datasets.
17. Annotation tools: ANTs image registration toolkit.

18. Measurement of inter- and intrarater variability: Intraclass correlation coefficients (ICC) were calculated for all qualitative categories to assess rating consistencies between both readers and methods. All distance and angle measurements were first evaluated regarding their normal distribution using a Shapiro-Wilk test. If a normal distribution was present, a paired sample t-test was applied to evaluate differences between readers, respectively methods. If measurements did not show a normal distribution, a Wilcoxon signed rank test was calculated and on all significant results a post-hoc Holm-Bonferroni test for multiple comparisons was performed.

Data Partitions

19. Sample size: A total of 91 participants were prospectively recruited and scanned with MRI including ZTE sequences. 11 ZTE image volumes were excluded due to poor image quality because of severe image inhomogeneity or metal hardware artifacts.
20. How data were assigned to partitions: Out of the 80 remaining data sets, 20 patient cases were selected as an evaluation cohort (= validation data set) and the remaining data was used to train and update the existing DL-model for pseudo-CT image synthesis from ZTE sequences (= training data set).
21. Level at which partitions are disjoint: n.a.

Model

22. Description of model: The deep learning model employed to convert MRI to pseudo-CT images is an adapted multi-task U-Net architecture. The model is trained in a supervised manner by using a co-registered CT as the reference ground truth to each input MR image. The three correlated tasks – (a) pseudo-CT image output (whole image region HU estimation); (b) bone mask segmentation; (c) bone region HU value estimation, were designed to provide enhanced focus on the bone regions in the image, maintaining the overall accuracy of image translation.
23. Software libraries, frameworks, and packages: Python 3.8, Tensorflow 2.10, ANTs library, ITK.
24. Initialization of model parameters: The model parameters were initialized to random numbers with the learning hyperparameters set as follows: Adam optimizer with initial learning rate of 0.0005, batch size of 25 images, number of training epochs set to 300.

Training

25. Details of training approach: The model training was driven by a linearly weighted sum of three loss functions, one corresponding to each task – (a) Weighted mean absolute error (wMAE) for the whole image regression; (b) Dice coefficient loss for bone segmentation; and (c) Weighted mean absolute error in the bone region. The co-registered CT image serves as the reference image for each MR image input.
26. Method of selecting the final model: The optimum model weights are selected based on the combined minima of the three loss functions, at a stage where the training and validation loss values are stable across training epochs and no oscillation or divergence is observed before the end of training.
27. Ensembling techniques: There were no ensembling techniques used in this model training. The final output is the predicted image from the selected single optimum model.

Evaluation

28. Metrics of model performance: The model was validated using mean absolute error (MAE) and structural similarity index metric (SSIM) values compared between the predicted pseudo-CT and the reference real CT images. The segmented bone regions from pseudo-CT and real CT were compared to access bone segmentation accuracy using Dice coefficient metric.
29. Statistical measures of significance and uncertainty: *P* values <0.05 were considered statistically significant. Intraclass correlation coefficients (ICC) estimates and their 95% confident intervals (CI) were calculated based on a mean-rating (k = 2), consistency-

agreement, 2-way mixed-effects model. ICC values less than 0.50 were considered poor, between 0.50-0.75 moderate, between 0.75-0.90 good, and above 0.90 as an excellent agreement.

30. Robustness or sensitivity analysis: Planned to be performed in future works of model development.
31. Methods for explainability or interpretability: Planned to be performed in future works of model development.
32. Validation or testing on external data: The trained model has been tested on external data from different sites for radiation treatment planning against CT based dose planning solutions.

Results

Data

33. Flow of participants: see Figure 1.
34. Demographic and clinical characteristics of cases: see Table 1.

Model performance

35. Performance metrics for optimal model: MAE pCT = 55 HU, MAE bone = 137 HU, Bone Dice = 0.91, pCT SSIM = 0.97.
36. Estimates of diagnostic accuracy and their precision: n.a.
37. Failure analysis of incorrectly classified cases: The predicted pseudo-CT images did not have gross misclassification/misestimation of any region. The regions corresponding to weak SNR of MR signal showed higher error in estimated values and thin bone regions were misclassified as soft-tissue. The improvement of overall accuracy of HU value estimation is scoped for future works.

Discussion

38. Study limitations: The authors acknowledge several limitations of this study. First, body masking for pseudo-CT remains challenging. This is mostly due to image inhomogeneity and attached external objects. To resolve this problem, additional background masking could be performed. In this study, the focus was however on deeper structures such as the pelvic bone and its adjacent soft tissues and body surface information was less important. Second, there were high-density areas in soft tissues that were occasionally classified as bone in pseudo-CT images. Those areas were mostly located in muscles around the pelvic bones and could imitate calcifications. This was due to the DL-model which was trained with enhanced focus on bone regions, thus showing a fuller appearing bone, but also resulting in some false bone classifications because of higher sensitivity towards high-density regions. In this context, it is important to notice that the ZTE data in this study were not enhanced by DL-reconstructions. Nowadays, DL-reconstruction solutions for ZTE images are available and have shown to enhance image quality. Therefore, higher DL-model performance in the generation of pseudo-CT images could be expected by employing DL-reconstructed ZTE images.
    Third, although HU and geometrical measurements were standardized, minor inconsistencies between readers during manual readout sessions might have persisted and impacted on overall pseudo-CT accuracy.
39. Implications for practice: Pseudo-CT images generated from MR sequences allow for high accuracy in evaluating bone without the need for radiation exposure. Radiological applications are broad and include assessment of inflammatory and degenerative bone disease or preoperative planning studies.

Other information

40. Registration number and name of registry: 2018-01752 (BASEC-ID, swissethics).
41. Accession of full study protocol: From the corresponding author on request.

42. Sources of funding: This study has received funding by the Gottfried und Julia Bangerter-Rhyner-Stiftung in form of a research grant.
